# Multi-Jet Electrospinning with Auxiliary Electrode: The Influence of Solution Properties

**DOI:** 10.3390/polym10060572

**Published:** 2018-05-23

**Authors:** Yu-Ke Wu, Liang Wang, Jie Fan, Wan Shou, Bao-Ming Zhou, Yong Liu

**Affiliations:** 1 State Key Laboratory of Separation Membranes and Membrane Processes, Tianjin Polytechnic University, Tianjin 300387, China; 13388096568@163.com (Y.-K.W.); wangliang@tjpu.edu.cn (L.W.); fanjie@tjpu.edu.cn (J.F.); 2 School of Textiles, Tianjin Polytechnic University, Tianjin 300387, China; zhoubaoming@tjpu.edu.cn; 3 Department of Mechanical and Aerospace Engineering, Missouri University of Science and Technology, Rolla, MO 65401, USA

**Keywords:** electrospinning, nanofibers, multiple jets, jet evolution

## Abstract

Multiple jets ejection in electrospinning has been a major approach to achieving a high production rate of ultrafine fibers, also known as nanofibers. This work studies the effect of solution parameters—including dielectric constant, polarity, conductivity and surface tension—on the jet number and jet evolution in the auxiliary electrode electrospinning approach. The results show that it is easier to generate 2–6 jets with short stable jet length (1.7–6.9 mm) under low voltage (5.03–7.13 kV) when solutions have higher dielectric constant (32.2–78.6) and larger surface tension (31.8–41.29 mN/m). The influence of solution properties on stable jet length and the influence of applied voltage to produce multiple jets are discussed in detail. This work provides a new perspective for understanding jet evolution and mass production of nanofibers in electrospinning.

## 1. Introduction

Studies of nanofibrous materials have dramatically increased over the past 20 years [1,2,3,4,5]. At a research-level production scale, electrospinning is the most widely used technique for nanofiber fabrication [6,7,8,9]. It can produce nanofibers with diameters in the range of tens of nanometers to few micrometers, which have large specific surface area and large porosity. The electrospun nanofibers have been applied in many fields, such as biomaterials [10,11], thermal responsive fibers [12,13], water treatment [14], actuators [15] and sensors [16,17]. However, traditional electrospinning techniques usually bring in single jets during spinning and a low yield of nanofibers (about 0.01–0.3 g/h), which limits the industrialization of nanofibers [18,19,20]. 

Some studies have proposed needleless electrospinning techniques to improve the production of electrospinning nanofibers [21]. Lu et al. [22] showed that the production throughput of needleless electrospinning was about 10 g/min, which was several thousand times higher than that of the traditional technique with a single needle as the spinneret. However, there are still some technological problems in the widespread promotion of needleless electrospinning technique, such as high voltage power supplies, rapid solvent evaporation and high costs. Electrospinning with multiple spinnerets is also an important route for the mass production of nanofibers [23,24,25]. However, due to the electrostatic interference between multiple spinnerets, especially when the number of spinneret increases to a certain size, some spinnerets cannot spin or drip in the form of droplets [26], resulting in uneven structure of the nanofiber membrane, perforation and other issues. In order to overcome these drawbacks, some modifications such as installing auxiliary electrodes to control jet instability, adjusting nozzle spacing and nozzle length [27,28] have been tested. However, some issues have still not been solved completely, such as the ejection of only a single jet from the spinneret that leads to a low production rate of nanofibers. In our previous work, a new electrospinning setup using a needle auxiliary electrode was proposed [29], which could produce 9–12 jets and had a throughput 7–10 times higher than that of traditional processes. Our previous study showed that it is possible to overcome the disadvantage of needleless electrospinning in which free liquid surface spinning has a high rate of solvent evaporation [29]. Each spinneret injected multiple jets and improved the nanofiber yield, while reducing the structural unevenness of fiber membrane. However, the effects of solution properties on auxiliary electrode electrospinning were still not clear.

In this work, we systematically explored the effect of solution properties—including dielectric constant, polarity, conductivity and surface tension—on the jet number and jet evolution in this auxiliary electrode electrospinning approach. The number of jets was also calculated under the same voltage from different solution systems, which was important for studying the mechanism of electrospinning multi-jet generation. Meanwhile, the stable jet length in all solutions was also studied.

## 2. Experimental Section

### 2.1. Material Preparation

Polystyrene (PS, molecular weight 335,000 g/mol), purchased from Sinopec Group of China and polyvinyl alcohol (PVA, molecular weight 83,000 g/mol) were used to prepare polymer solutions. When investigating the influence of concentration on the morphology of electrospun fibers, the researchers found that when the solution of concentration *C* and intrinsic viscosity [η] is a certain value, the morphology of electrospun fibers is basically similar for similar polymer solutions, irrespective of the molecular weight of the polymer. The [η]*C* is called the Berry number [30], and the value can be used to characterize the degree of polymer chain entanglement. The intrinsic viscosity (intrinsic visco) [η] is one of most common representation of the viscosity of the polymer solution. It is defined as the ratio concentration or the logarithmic viscosity when the concentration of the polymer solution tends to zero. When the same solvent and temperature are kept, the relationship between the relative molecular mass and the intrinsic viscosity of the polymer are determined by the Mark–Houwink equation:

[η] = *KM*^α^ (1)

In a certain relative molecular weight range, *K* and α are Mark–Houwink constant [31], and these constants are independent of the relative molecular weight. The different *K* and α are shown in Table 1. According to the Mark–Houwink equation, the molecular weight can be used to calculate the intrinsic viscosity [η] of a certain polymer in a solvent and the polymer powder was dissolved in various solvents to prepare the polymer solution at a fixed berry number of 9. In order to compare the results, the properties of solutions (surface tension, conductivity and dielectric constant) were measured, as shown in Table 2.

### 2.2. Measurement and Characterization

The conductivity and surface tension of all solutions were measured by using a conductivity meter (DDS-12DW, Shanghai Bante Co. Ltd., Shanghai, China) and an interfacial tension meter (JYW-200A, Chengde Jinhe Co. Ltd., Chengdu, China), respectively, at 25 °C. The trajectory of electrospun jet was observed by a digital camera (FUJIFILM FinePix HS11, Japan). The average number of jets was recorded five times by the digital camera and other conditions were kept: ESD as 2 cm, injection speed as 2 mL/h, spinning distance as 15 cm, and the applied voltage at 6, 7, 8, 9, 10, 11 and 12 kV.

### 2.3. Experimental Setup

**Figure 1:**
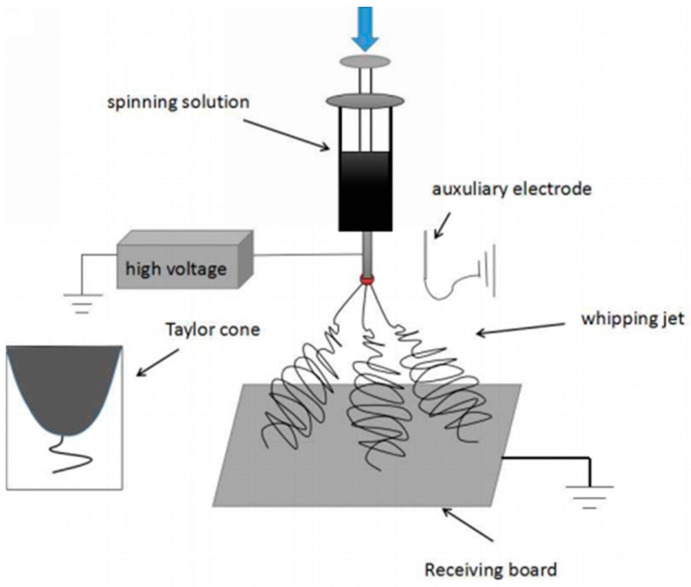
Experimental setup (a needle auxiliary electrode next to the spinneret).

As shown in Figure 1, a modified electrospinning device with a needle auxiliary electrode parallel to the spinneret was employed in this study. The diameter of the spinneret and auxiliary electrode were 8 and 5 mm, respectively. The spinning distance between the spinneret and the collector was 15 cm. Other parameters could be adjusted according to experiments, e.g., the distance between the auxiliary electrode and the spinneret (labeled as ESD) at 3 cm, injection speed at 2 mL/h, and applied voltage at 4–10 kV.

## 3. Results and Discussion

### 3.1. Effect of Solution Properties on Jet Evolution

**Figure 2:**
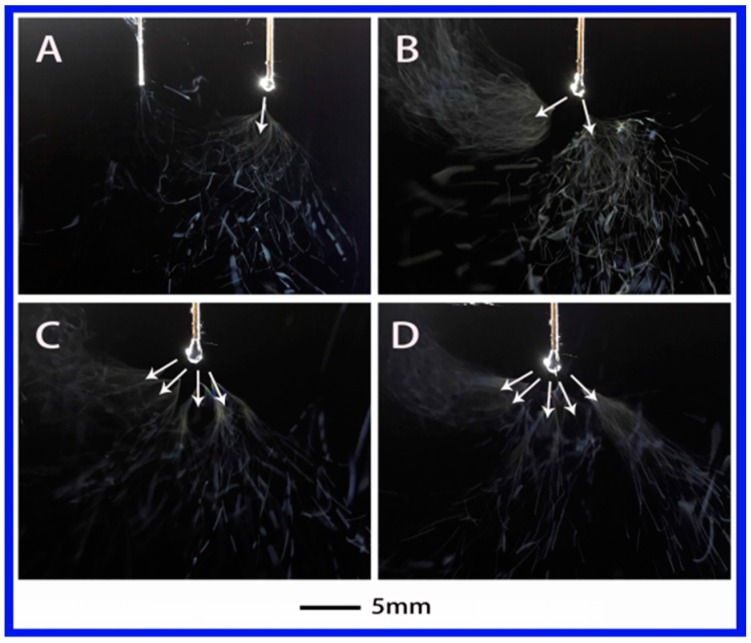
The jet motion of PVA (polyvinyl alcohol)–water solution under different applied voltages: (**A**) 4.8 kV, (**B**) 5.62 kV, (**C**) 6.75 kV, and (**D**) 7.13 kV. The distance between the auxiliary electrode and the spinneret (ESD) of 3 cm and injection speed of 2 mL/h.

In general, the solution properties including dielectric constant, surface tension and conductivity are the main factors affecting electrospinning process. In order to investigate the effect of dielectric constant on the jet, five solutions with different polarities were studied as shown in Table 1. In this work, the injection flow rate was fixed to 2 mL/h due to a high fiber yield of 7–10 times larger than that of traditional electrospinning under the same flow rate [29]. Figure 2 shows the jet motion of PVA–water solution under different applied voltages. It was clear that the jet of PVA–water solutions underwent a series of unstable states after a steady state. When the applied voltage was 4.8 kV, the primary jet appeared first from Taylor cone, followed by the jet whipping. When the voltage was increased to 5.62 kV, two jets emerged, as illustrated in Figure 2B. When the applied voltage was further increased to 6.75 and 7.13 kV, multiple jets (four and five) were formed, as shown in Figure 2C,D. In this case, water is a strong polar solvent. This means the electrolytes can be easily dissociated into ions in the PVA–water solution and enhance its conductivity, thus increasing the surface charge density on the jet. This may be the reason for multiple jets formation from PVA–water solution. Here, the primary jet underwent a short distance of the steady tensile effect and then entered the unstable region due to the presence of an applied electric field and the surface charge of the jet.

**Figure 3:**
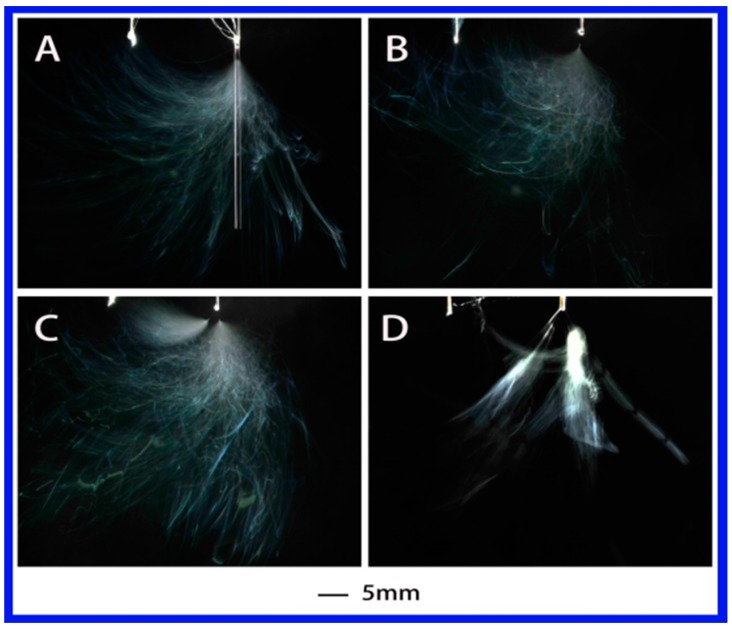
Jet motion of PS–DMF (polystyrene–dimethylformamide) solution under different applied voltages: (**A**) 4.63 kV, (**B**) 4.52 kV, (**C**) 5.08 kV, and (**D**) 6.55 kV with ESD of 3 cm and injection speed of 2 mL/h.

Figure 3 shows the evolution of jets during electrospinning via a needle auxiliary electrode involved using PS–DMF (dimethylformamide) solution under different applied voltages. The single jet underwent stable jet stage for the initial few seconds after it emerged from the Taylor cone when applied voltage was 4.63 kV. As the voltage increased, the number of jets became 2 and 3 at 5.08 and 6.55 kV, respectively. The general evolution was similar to the PVA–water solution, but the total number of jets decreased by about half and the formation of multiple jets was not as stable as the PVA–water solution, which might be associated with the dielectric constant. As indicated in Table 2, from PVA–water solution to PS–DMF solution, the dielectric constant decreased from 78.36 to 36.71, which is also about half. 

**Figure 4:**
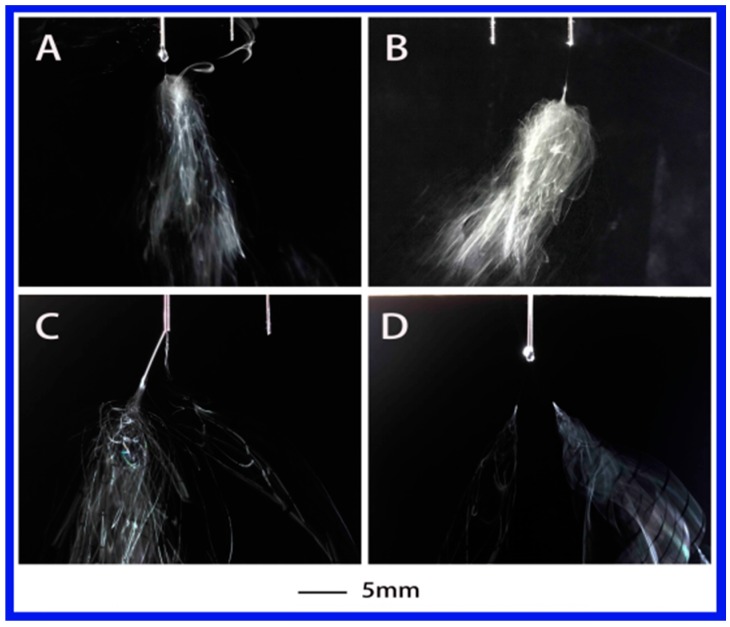
The jet motion of PS–NMP (*N*-methyl-2-pyrrolidinone) solution under different applied voltages: (**A**) 4.38 kV, (**B**) 4.45 kV, (**C**) 5.03 kV, and (**D**) 5.32 kV with ESD of 3 cm and injection speed of 2 mL/h.

Further decrease of dielectric constant to 32.2 with the PS-NMP (*N*-methyl-2-pyrrolidinone) solution, the corresponding jet evolution is presented in Figure 4. As expected, the formation of the primary jet shared similarities with the above two cases. A single jet appeared when the applied voltage was 4.45 or 4.38 kV, followed with jet instabilities. Multiple jets (but no more than two) were observed when the voltage was increased to 5.03 and 5.32 kV. Meanwhile, the multi-jet state was much less stable compared with the above two solutions.

**Figure 5:**
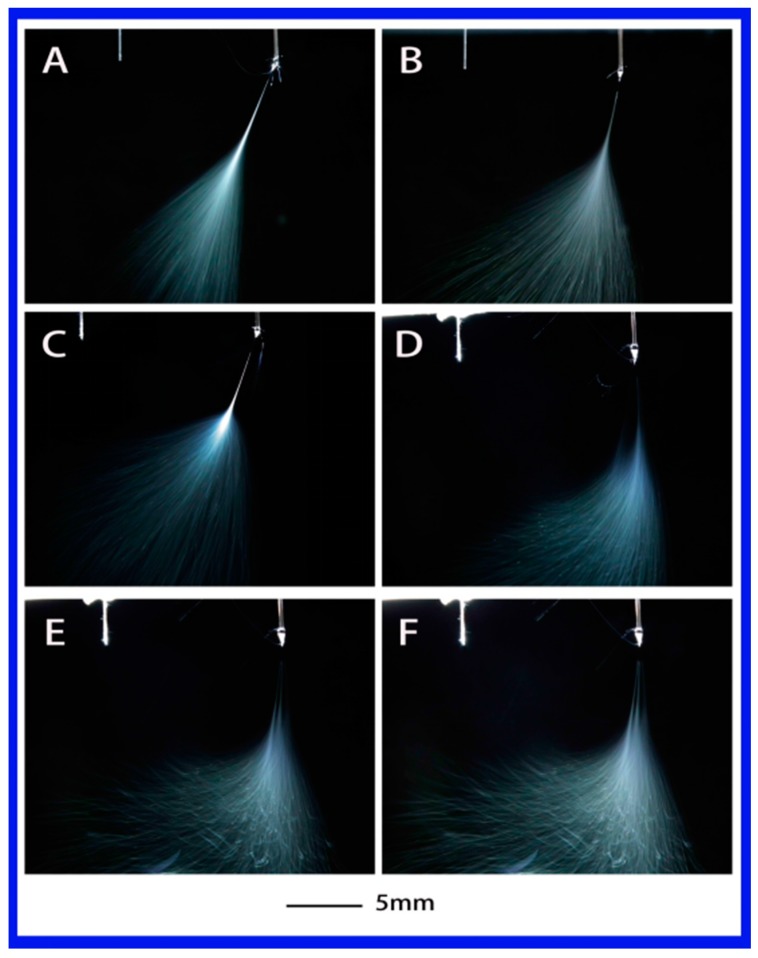
The jet motion of PS–THF (tetrahydrofuran) solution under different applied voltages: (**A**) 6 kV, (**B**) 6.2 kV, (**C**) 6.56 kV, (**D**) 7 kV, (**E**) 7.2 kV, and (**F**) 7.5 kV with ESD of 3 cm and injection speed of 2 mL/h.

The influence of dielectric constant (or polarity) can be directly seen among the above three strong polar solutions. When a medium polarity solution (PS–THF (tetrahydrofuran), with dielectric constant of 7.58) was used, its jet evolution was recorded and shown in Figure 5. It is clearly evident that no matter what the applied voltage is, it is difficult to obtain more than one jet during the electrospinning. Also, the jet from this solution has a long stable jet length and shows a regular jet differs from previous solutions, which exhibits buckling jets like a cloud. Additionally, the shape of single jets are similar to each other, as shown in Figure 5A–F, although the whipping area varies.

**Figure 6:**
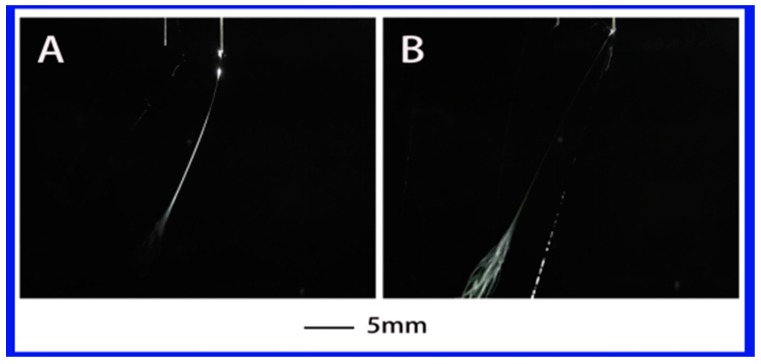
The jet motion of PS–chloroform solution under different applied voltages: (**A**) 6.34 kV, (**B**) 7 kV. ESD of 3 cm and injection speed of 2 mL/h.

When the dielectric constant is reduced further to 4.81 (where the PS–chloroform solution was used), the solution became non-polar. Similar to the PS–THF solution, only a single jet was obtained, as shown in Figure 6. In this case, the charges were mainly concentrated on the surface of the droplet under a high applied voltage [32]. Under the action of the electric field, the surface charge caused the flow on surface of droplet, thus forming a jet. It is obvious that the generation of jet is closely related to the charge on the surface of the droplet. It was difficult to produce multiple jets with this solution. It might be attributed to the relatively low dielectric constant and the polyelectrolyte behavior of chloroform, causing a low charge density in the jet. Thus, the properties of the solution such as the dielectric constant and the electrical conductivity directly determine the ability of the droplet to accumulate charge. In other words, the properties of the solution have a critical effect on the generation of multiple jets.

### 3.2. Effect of Solution Properties on Stable Jet Length

**Figure 7:**
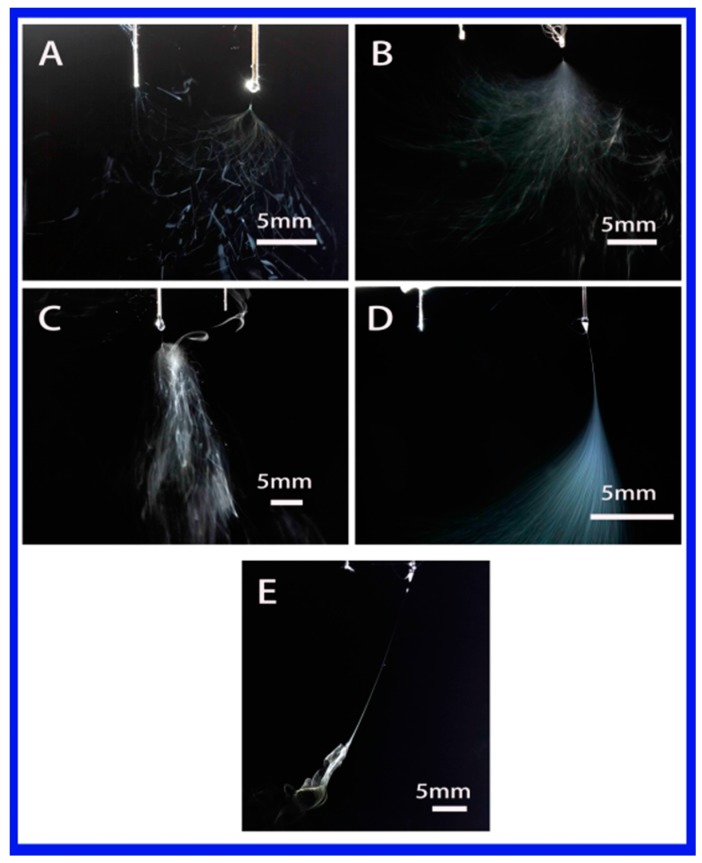
Photographs showing the single jet during electrospinning via an auxiliary electrode (Solvent (**A**–**E**): water, DMF, NMP, THF and chloroform).

In this section, the effect of solution properties on stable jet length was investigated. Stable jet lengths of single jet for all solutions were firstly compared, as shown in Figure 7. All the polymer jet ejected from the top of the Taylor cone moved in the opposite direction of its electrode. The jet usually went through a short distance from the stable movement first and then entered the unstable movement stage. From Figure 7 and Table 2, it can be found that the jet motion was observed with a cloud of jets and a shorter stable jet length when a solvent with high dielectric constant, e.g., water, DMF and NMP, were used. The lengths (1.56–6.9 mm) of stable jets in high dielectric constant solvents were much shorter than those in low dielectric constant solvents. For instance, the lengths were 7.53 and 21.23 mm in THF and chloroform, respectively. Stable jet length increases with the decrease in dielectric constant of solvent, as shown in Table 3, which can be used to conclude that the dielectric constant of the solvent plays a dominant role in the length of stable jets.

**Figure 8:**
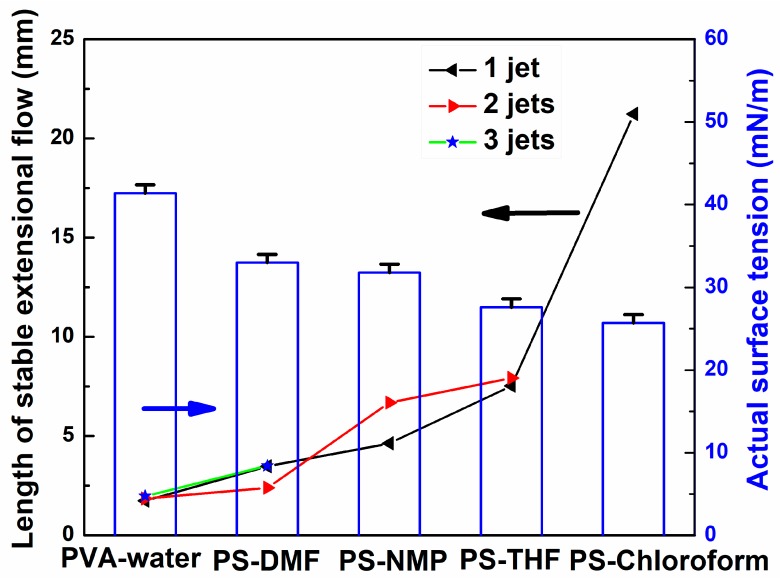
The influence of surface tension on the length of stable jet for different solutions.

The average length of multiple jets is also influenced by the surface tension of solution. Figure 8 shows the influence of surface tension on the length of stable jets for different solutions. The average length of stable jets of all solutions and their actual surface tension were measured. It can be seen that the larger the actual surface tension of solution, the shorter is the length of stable jet generated. 

### 3.3. Effect of Solution Properties on Applied Voltage to Produce Jets

**Figure 9:**
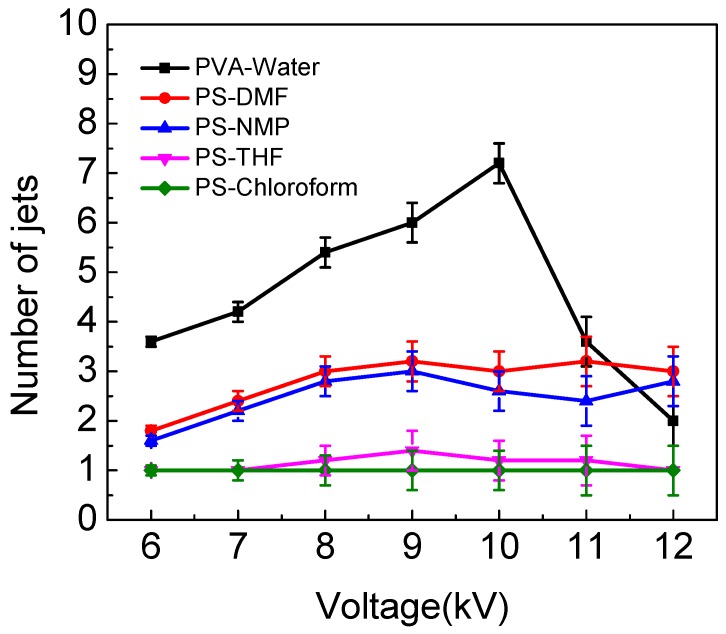
The influence of applied voltage on the number of jets for different solutions.

In order to better understand the effect of different solution properties on the number of multiple jets, other conditions were kept at the ESD as 2 cm, injection speed as 2 mL/h, spinning distance as 15 cm, and the applied voltage was controlled at 6, 7, 8 9, 10, 11 and 12 kV. As Figure 9 shows, when the applied voltage is 6 kV, the number of jets for PS–NMP solution was calculated and the data was recorded at intervals of five. The results were 2, 2, 2, 1 and 1. This means this solution produced two jets three times and a single jet two times; the average number of jets in PS–NMP solution was 1.6. At the same time, the numbers of jets in PS–DMF solution were two jets four times and a single jet once at 6 kV; the average number of jets was 1.8. As the results in Figure 9 show, the average number of jets in PVA–water is the highest among all solutions. It can be seen that multiple jets could be produced when water, DMF and NMP were used as solvents. Occasionally, it could produce two jets with the THF solution, and it could only produce a single jet when chloroform was the solvent. It can be observed that the greater the conductivity of a solution, the greater the number of jets generated. In our previous work [29], FEM software ANSYS was applied to calculate the electric field distribution around the spinneret to explain how multiple jets were produced by auxiliary electrode electrospinning. We found that the introduction of the auxiliary electrode enhances the electric field strength at the spinneret tip. This may be the reason that multiple jets form as it appears to be a critical parameter to ensuring effective jet formation. In fact, a solution’s property, such as conductivity, is closely related to electric field strength around the spinneret. With the solution conductivity increasing, the surface charge of the jet increased, further enhancing the splitting capacity of the droplet and resulting in larger electrostatic repulsion. Under the same applied voltage, a solution with high conductivity can produce more jets than solution with low conductivity. This theory is consistent with previous experimental results.

### 3.4. Effect of Solution Properties on Fiber Morphology

**Figure 10:**
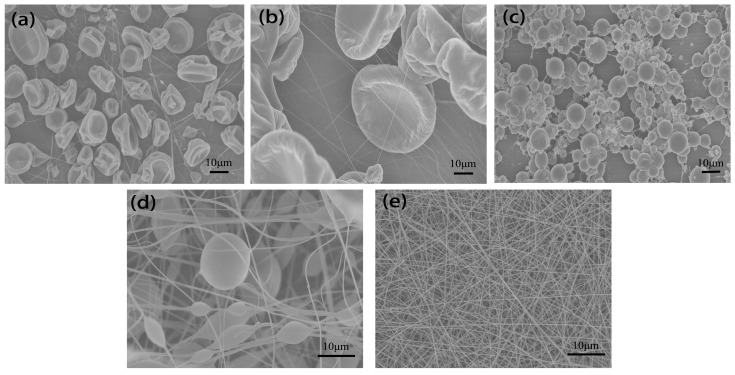
SEM images of fibers and beads electrospun from various solutions: (**a**) PS–chloroform, (**b**) PS–THF, (**c**) PS–NMP, (**d**) PS–DMF, (**e**) PVA–water with ESD of 3 cm, applied voltage of 9 kV, spinning distance of 15 cm and injection speed of 2 mL/h.

Representative SEM images of nanofibers are shown in Figure 10 with different solutions. Figure 10a,b show that the structure consisted of fine fibers and cup-shaped beads, typically in the range of 10–40 μm when THF and chloroform were used as solvents. Figure 10c was obtained using PS–NMP solution, it can be clearly seen that the structure consisted of many beads, which are much smaller in size (1–10 μm) compared to those obtained with THF and chloroform. When DMF was used as solvent, there were less beads and highly fibrous structure was observed as indicated in Figure 10d. In contrast to these beads, the morphology obtained with PVA–water solution consisted of fine fibers without beads (Figure 10e), indicating that the transition from bead to fiber may relate to the polarity of solution.

## 4. Conclusions

The property of solution is an important factor in jets evolution of electrospinning via an auxiliary electrode. Some properties such as dielectric constant and surface tension were found to play a dominant role in determining jet behavior during the electrospinning. Two classes of solutions were identified based on the development of jet instabilities. The first class of solutions is characterized by producing no more than one jet with a long stable jet length (7.53–21.3 mm), such as PS–THF and PS–chloroform solutions, which have low dielectric constant and surface tension. However, the second class of solutions of PS–DMF, PS–NMP and PS–water could easily produce multiple jets (≥2) with short sable jet length (1.73–6.9 mm). The results show that polymer solutions with higher dielectric constant and surface tension will produce more jets with short stable jet length at low applied voltage.

## Figures and Tables

**Table 1:** Mark–Houwink constants for the solutions used in this study.

Polymer	Solvent	Polymer Molecular Weight	K*10^3^ (mL/g)	α	Intrinsic Viscosity [η]	Concentration (*C*) (g/mL)	[η]*C*	Polarity
PVA	Water	83,000	20	0.76	109.53	0.082	9	Strong
PS	DMF	335,000	31.8	0.603	68.238	0.132	9	Strong
PS	NMP	335,000	12	0.72	114.08	0.079	9	Strong
PS	THF	335,000	11	0.725	111.44	0.081	9	Semi
PS	Chloroform	335,000	7.16	0.76	113.23	0.079	9	Non

Notes: *K* and α are Mark–Houwink constant. PVA: polyvinyl alcohol; PS: polystyrene; DMF: dimethylformamide; NMP: *N*-methyl-2-pyrrolidinone; THF: tetrahydrofuran.

**Table 2:** Solutions used in this study.

Solution	Actual Surface Tension (mN/m)	Maximum Surface Tension (mN/m)	Conductivity (μs/cm)	Dielectric Constant ε
PVA–water	41.39	49.48	0.05	78.36
PS–DMF	32.99	40.3	0.041	36.71
PS–NMP	31.8	38.98	0.036	32.2
PS–THF	27.6	34.3	0.0252	7.58
PS–chloroform	25.7	28.9	0.0198	4.81

**Table 3:** The length of stable jet of all solutions. The notation “-“ means the solution cannot produce jets.

Solution	Single jet (mm)	Two jets (mm)	Multiple jets (mm)
PVA–water	1.73	2.13 1.56	2.13 1.43 2.34
PS–DMF	3.47	3.02 1.76	4.2 4.18 2.12
PS–NMP	4.63	6.9 6.45	-
PS–THF	7.53	7.54 8.3	-
PS–chloroform	21.23	-	-
